# Dynamic spatiotemporal trends of imported dengue fever in Australia

**DOI:** 10.1038/srep30360

**Published:** 2016-07-27

**Authors:** Xiaodong Huang, Laith Yakob, Gregor Devine, Francesca D. Frentiu, Shiu-Yun Fu, Wenbiao Hu

**Affiliations:** 1 School of Public Health and Social Work, Institute of Health and Biomedical Innovation, Queensland University of Technology, Brisbane, Queensland, Australia; 2 Department of Disease Control, London School of Hygiene and Tropical Medicine, London WC1E 7HT, UK; 3 Mosquito Control Laboratory, QIMR Berghofer Medical Research Institute, Brisbane, Queensland, Australia; 4 School of Biomedical Sciences, Institute of Health and Biomedical Innovation, Queensland University of Technology, Brisbane, Queensland, Australia; 5 Department of Nursing, College of Medicine, Fu Jen Catholic University, New Taipei City, Taiwan

## Abstract

Dengue fever (DF) epidemics in Australia are caused by infected international travellers and confined to Northern Queensland where competent vectors exist. Recent analyses suggest that global trade and climate change could lead to the re-establishment of *Ae. aegypti* across the country and promote the spread of dengue nationally. This study aimed to describe the dynamic spatiotemporal trends of imported DF cases and their origins, identify the current and potential future high-risk regions and locate areas that might be at particular risk of dengue transmission should competent mosquito vectors expand their range. Our results showed that the geographical distribution of imported DF cases has significantly expanded in mainland Australia over the past decade. In recent years, the geographical distribution of source countries of DF has expanded from the Pacific region and Asia to include Africa and the Americas. Australia is now exposed to dengue importations from all of the regions involved in the current global pandemic. The public health implications of a range expansion of dengue mosquito vectors are severe. Enhanced mosquito surveillance in those areas that have high imported cases is called for to reduce emerging threats from this globally expanding pathogen.

In terms of both morbidity and mortality, dengue fever (DF) is the most important arboviral disease in the tropics and subtropics^1^. The geographic distribution of dengue virus has rapidly expanded since World War II^2^. Over 190 countries have the vector mosquitoes *Aedes aegypti* and *Ae. albopictus* and 150 countries are now reported to have autochthonous transmission^3^. An estimated 390 million people are infected by dengue virus each year worldwide^4^. The clinical presentation of dengue infections vary from inapparent, to dengue haemorrhagic fever and potentially fatal dengue shock syndrome^5^. No licensed vaccination or specific treatment against dengue virus is available^4^. Globalization and climate change have been implicated in the expanding geographic ranges of both dengue virus and dengue vector mosquitoes. International travel is particularly relevant for seeding and reseeding outbreaks in regions of the world that are currently situated at the fringes of endemicity^6^.

In Australia, the first records of DF date from 1873 and these were followed by a spate of epidemics in Queensland (QLD), northern Western Australia (WA), northern New South Wales (NSW) and the Northern Territory (NT) which lasted until about 1943^7^. Historically recorded DF incidences were much higher than current dengue incidence in Australia including major outbreaks in Charters Towers in 1897 and in Brisbane in 1905; and high mortalities associated with dengue haemorrhagic fever were reported during this period^7^. These records demonstrate the extensive range that was suitable for dengue transmission in Australia’s recent past. Currently, dengue epidemics are restricted to northern Queensland where *Ae. aegypti* breed. Exactly why the range of the vector has shrunk from its historic distribution across QLD, NSW, WA and NT is unclear, but it is probably related to post-war changes in water storage patterns, a period of intensive mosquito control, and competition from native mosquitoes^8^.

Importantly, recent analyses suggest that global trade and the increasing temperature and unreliable rainfall patterns expected from climate change could lead to the re-establishment of *Ae. aegypti* across the country^8^,^9^,^10^,^11^. Moreover, a secondary dengue vector mosquito *Ae. albopictus* has been found on islands of the Torres Strait and further threatens the spread of dengue across mainland Australia^12^,^13^.

Of more immediate concern is the increased frequency of dengue outbreaks in northern QLD that have occurred following the opening of an international airport in Cairns in 1984^14^. Concordantly, here has been an increase in dengue importations in general. Dependent on the location and the time of year, these importations risk sparking autochthonous events. Autochthonous transmission in northern QLD is now practically an annual event–with 1782 local cases between 2005–2014 including a particularly large epidemic in 2009 involving 915 cases^15^. Although Australia is presently considered a non-endemic country^7^, this status is threatened by the permissive conditions of north QLD^16^,^17^. A better understanding of the magnitude and pattern of imported DF cases and identification of the countries of acquisition is necessary for public health services to monitor dengue transmission risks in currently vulnerable locations and in those threatened by invasions or range expansions of key vectors. This study aimed to examine the spatiotemporal pattern and origins of imported DF cases and identify potential risk regions affecting dengue transmission in Australia.

## Results

### Spatial and temporal distribution of imported DF cases

**Figure 1:**
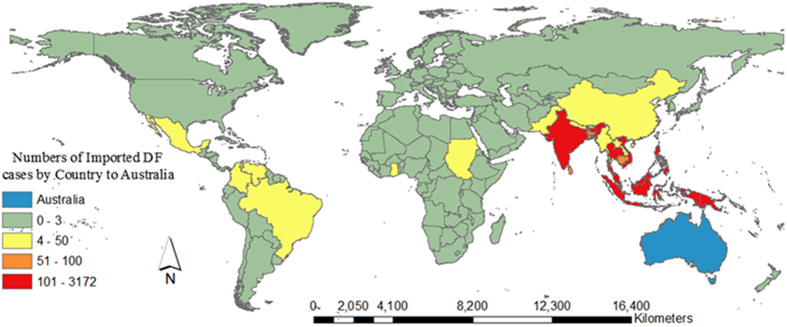
The spatial distribution of imported DF cases by country of acquisition during 2004–2013 in Australia^36^.

**Figure 2:**
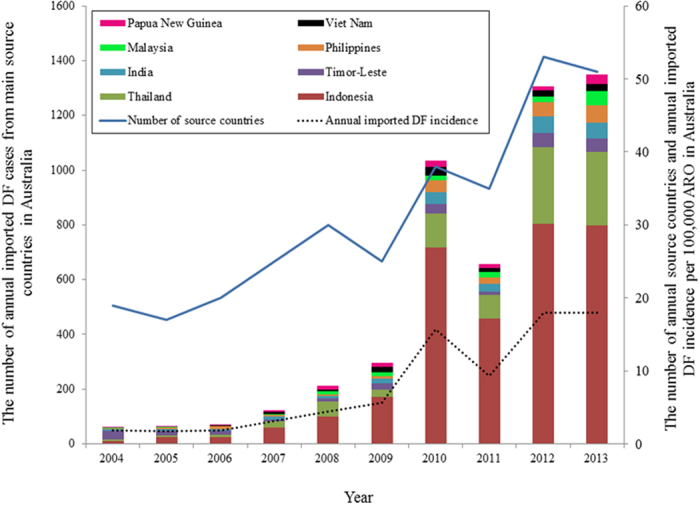
Number of source countries and imported DF incidence between 2004 and 2013 (Annual imported DF incidence per 100,000 ARO). Blue line and dashed line showed the trends of the number of annual source countries of DF and annual imported DF incidence between 2004 and 2013, respectively. The bars showed the number of annual imported DF cases from main countries during 2004 to 2013.

Figure 1 shows the spatial distribution of imported DF cases by country of acquisition during 2004–2013. During this time period in Australia, 5,943 imported cases were recorded, with the highest annual imported DF cases in 2013 (1,583 cases). There was an increased trend in annual imported DF incidence with an average incidence of 7.78 per 100,000 Australian residents returning from overseas (ARO) over the study period. The lowest annual imported DF incidence was reported in 2005 (1.65 per 100,000 ARO) and the highest annual imported DF incidence (17.75 per 100,000 ARO) was observed in 2013 (Fig. 2). The top five countries of acquisition were Indonesia (3172 cases), Thailand (879 cases), Timor-Leste (252 cases), India (243 cases) and the Philippines (222 cases) (Fig. 2). The annual number of ARO steadily increased from 4,494,670 people in 2004 to 8,939,720 people in 2013 in Australia.

### Seasonality of imported DF

**Figure 3:**
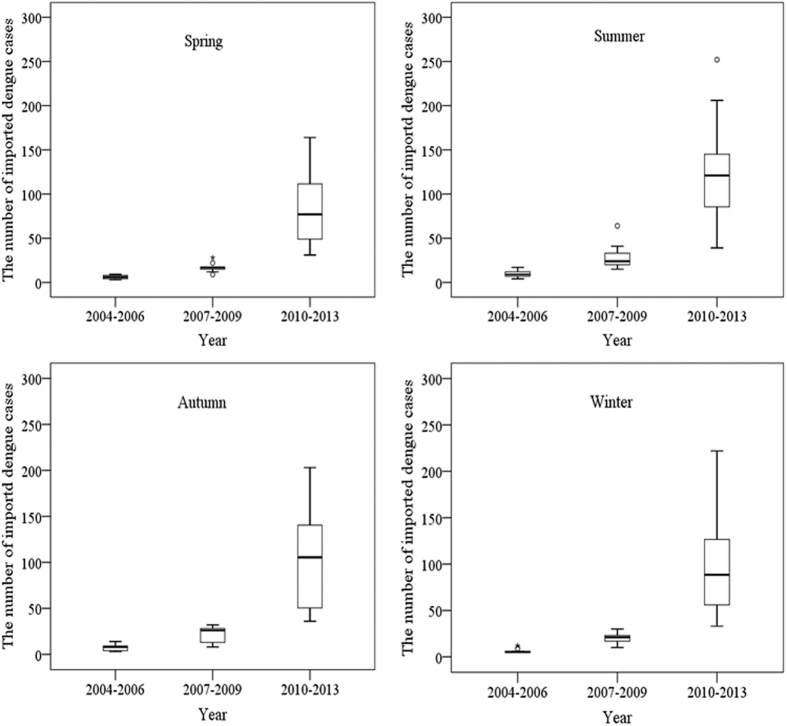
Boxplots of monthly imported DF cases across four seasons during the three study periods: 2004–2006, 2007–2009 and 2010–2013.

**Figure 4:**
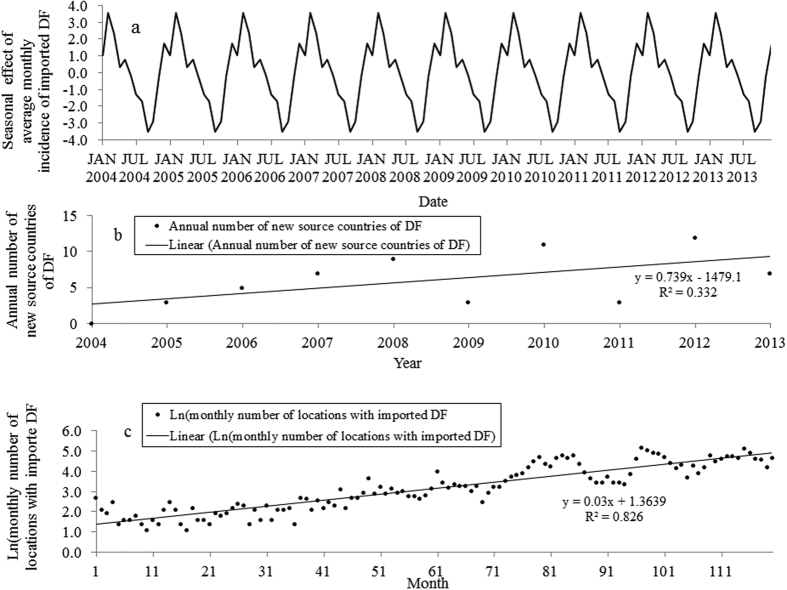
(**a**) The pattern of the seasonal effect of imported DF over the study period in Australia. (**b**) Scatter plot with linear regression line for annual number of new countries of acquisition against time (year). (**c**) Scatter plot with linear regression line for the monthly number of the postal locations that received imported DF cases against time (month).

There was an increasing trend of seasonal variability of imported DF cases from 2004 to 2013 (Fig. 3). Nationally, summer (December–February) had the highest average of monthly imported cases (mean = 61.3 cases per month) and spring (September–November) had the lowest average of monthly imported cases (mean = 39.2 cases per month). Among the three periods of 2004–2006, 2007–2009 and 2010–2013, the highest mean of monthly imported DF cases for the four seasons were observed during the period 2010–2013, with the monthly means of 80.8 cases (range 31–164 cases) in spring, 124.3 cases (range 39–252 cases) in summer, 103.8 cases (range 36–203 cases) in autumn (March–May) and 98.3 cases (range 33–222 cases) in winter (June–August) (Fig. 3). However, the seasonal decomposition analysis showed that the pattern of seasonal effects of average monthly incidence of imported DF was consistent throughout the study period (Fig. 4a). The peak seasonal effect on average monthly incidence occurred in February and the lowest seasonal effect was observed in September.

### Spatiotemporal trend of imported DF cases

**Figure 5:**
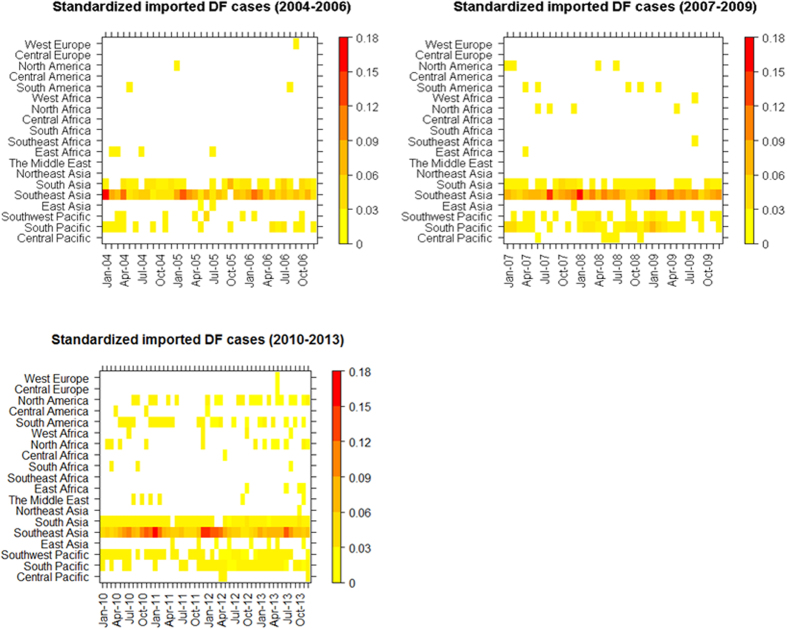
Heat maps of standardized monthly imported cases from Pacific regions, Asia, Africa, Europe and the Americas. The monthly imported DF cases were standardized by the national totals of imported DF cases each year.

There were increasing linear trends in the annual number of new source countries of DF and the monthly number of postal locations with imported DF (Fig. 4b,c). From the simple linear regression models, the annual number of new countries of acquisition increased by 0.739 per year (p = 0.081) (Fig. 4b). The log-transformed monthly number of postal locations where imported cases were observed in Australia increased by 0.03 per month (p < 0.000) (Fig. 4c). The heat map shows the spatiotemporal patterns of standardized monthly imported cases from the Pacific region and from the four continents over the three periods (Fig. 5). The geographical distribution of source countries of DF has substantially expanded from the Pacific region and Asia to also include Africa and the Americas in more recent years. In Asia, Southeast Asia was the most important region of acquisition related to the highest proportion of annual imported cases through the study period. A total of 53.4% of imported cases in Australia were from Indonesia with the highest case numbers reported in 2012 and 2013. Following 2010, the Middle Eastern countries, such as Saudi Arabia and United Arab Emirates, were also sources of imported DF cases into Australia. The South and Southwest Pacific, including Papua New Guinea and Fiji, were also main regions of acquisition. In Africa, source regions geographically expanded from East Africa to other parts of Africa, particularly North Africa, during 2010–2013. The frequency of reported DF cases imported from the Americas has also increased since 2010. Imported DF cases from Central America were only found in 2010 and 2012. From Europe, only three cases were reported from Central and Western Europe over the period of the study (Fig. 5).

### Spatiotemporal mapping of imported DF cases

**Figure 6:**
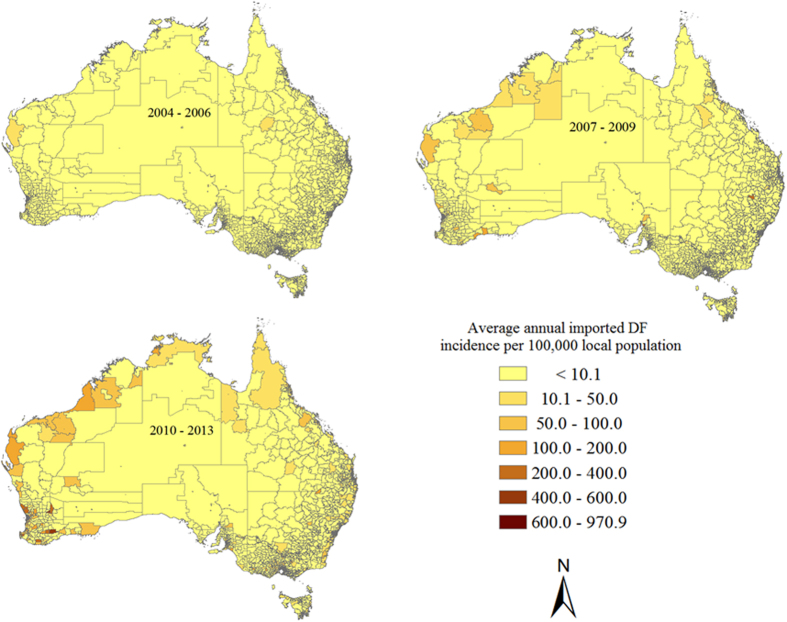
Spatiotemporal distribution of average annual imported DF incidence per 100,000 local population during the three periods: 2004–2006, 2007–2009 and 2010–2013^36^.

There was substantial spatiotemporal variation in average annual local imported DF incidence across Australia during the three periods (Fig. 6). During 2004–2006, the average annual imported DF incidence rates ranged from 1.7 to 176.1 per 100,000 local population among 159 postal locations across Australia. The highest average annual imported DF incidence was detected in Darwin (176.1 per 100,000 local population), which is the capital city of NT in the northern tip of Australia, followed by a second location in NT (136.6 per 100,000 local population). During 2007–2009, the average annual imported DF incidence varied between 1.7 and 699.3 per 100,000 local population in 371 postal locations across Australia, of which 15 locations had an average annual imported DF incidence of more than 100 per 100,000 local population. Those locations were found in QLD, South Australia (SA), NT, WA and NSW. Tasmania, an island state located 240 kilometres to the south of the Australian mainland, recorded its first appearance of imported dengue cases in 2010. During 2010–2013, the average annual imported DF incidence ranged from 1.9 to 970.9 per 100,000 local population among 1113 postal locations across Australia, with 109 locations reporting an average annual imported DF incidence of more than 100 per 100,000 population. These were particularly common in WA (Fig. 6).

### Spatial autocorrelation and risk analysis of imported DF cases

**Figure 7:**
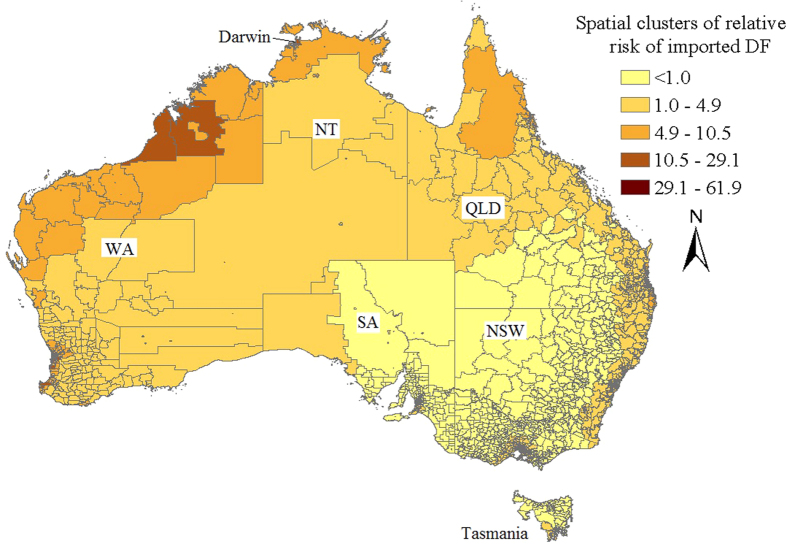
Spatial clusters of low and high relative risks of imported DF across Australia after adjustment for the local SEIFA during 2004–2013^36^.

Statistically significant spatial autocorrelation (Moran’s *I*: 0.063, *P* < 0.001) was demonstrated in local imported DF incidences during the period 2004–2013. Local SEIFA was positively and significantly associated with local imported DF cases. The Bayesian spatial Poisson model showed an increase in mean local imported DF cases of 0.25% (95% credible interval: 0.18–0.32%) for 1 unit increase in the socio-economic index for area (SEIFA) at postcode-level between 2004 and 2013. Spatial clusters of high relative risk of imported DF identified by the CAR model after adjustment for the local SEIFA were mainly found in the western and northern parts of Australia (Fig. 7).

## Discussion

This study explored the spatiotemporal distributions of imported DF cases across Australia and the countries of acquisition. Spatial clusters of high imported DF risk were identified for the purpose of highlighting areas at particular risk of dengue epidemics in Australia and also those that might be most threatened by expansions in the ranges of *Ae. aegypti* or *Ae. albopictus*.

A strongly seasonal pattern of dengue importations was apparent. The highest numbers of imported dengue cases occurred in summer which includes the Christmas and New Year vacation period in Australia when international travel peaks. After adjustment for the monthly number of Australian residents returning from overseas, the peak monthly imported DF incidences remained in the summer (February). This reflects the seasonality of dengue epidemics in the key countries of origin^18^,^19^. Understanding the seasonality of risk is important to Australia’s health and quarantine services that might enhance surveillance or increase their public health messaging at corresponding times of the year.

An increasing number of source countries of DF were responsible for the expanding spatial distribution of imported DF cases during the period 2004–2013 in Australia. Although Asia (particularly Southeast Asia^17^,^20^) and the Pacific region have been traditionally recognized as the major sources of imported DF cases in Australia, a dramatic expansion in the number of source regions of imported DF cases is evident from this current analysis. Central Pacific, West Asia, North Africa and South America are now important contributors of imported DF, reflecting increased international travel and corresponding with a global expansion in dengue endemicity. This poses a considerable challenge for future dengue prevention and control in Australia.

An increased number of postal locations reporting imported DF was observed in the study. Interestingly, our estimated spatial clusters of high local imported DF incidences displayed some of the same patterns historically observed for the dengue vector^8^, further justifying the close monitoring of these regions. Improved mosquito surveillance in these prone areas, in response to virus importations, would facilitate a risk assessment regarding local transmission and help prioritise responses at the state level. While certain risk factors associated with dengue vector presence will likely be ameliorated when compared with historic distributions (e.g. improved housing), other factors such as climate might offset these improvements (e.g. cause an increase in urban water storage behaviour)^21^. High-risk clusters were identified in the northern, western, eastern and south-eastern parts of Australia. The three maps of the average annual local imported DF indicated that high imported DF incidences were primarily concentrated in the northern tip of Australia during the period 2004–2006 but have subsequently expanded across the country. This may reflect changed travel habits over the past decade with increased rates of Australians visiting Asia^17^. Higher local SEIFA, reflecting higher local socio-economic status was also significantly associated with an increase in local imported DF incidences in the study. Travel and trade between countries is typically considered the most important risk factor for the spread of the dengue virus and vector mosquitoes from endemic countries to non-endemic countries^22^,^23^,^24^ and these behaviours are more associated with affluent individuals. These richer SEIFAs are also likely to be foci of travel to Australia by foreign nationals.

It has been noted that dengue infections may be subclinical (or inapparent) but can still be transmitted to mosquitoes and then to humans^4^,^25^; and this points to an important limitation of the current study. Data used in this analysis were on laboratory confirmed cases only but it is likely that a substantial proportion of infected individuals were asymptomatic or suffered only mild symptoms–and therefore did not seek medical care. That said, these missing data would not be anticipated to skew or affect the identified hotspot distributions because they are likely to be randomly distributed across all locations.

## Conclusion

The incidence of DF appears to be increasing in travellers returning from Southeast Asia, Africa and the Americas. The geographical distribution of imported DF cases has significantly expanded in mainland Australia over the past decade along with the number of countries of acquisition. Of concern is the fact that the current distribution of imported cases in Australia closely matches the historic range of the dengue vector, *Ae. aegypti*. Understanding of the global origins of imported dengue as well as identifying local Australian import hotspots is an important driver for targeting efforts in mosquito surveillance and control. This will help mitigate the future spread of this globally expanding pathogen and its vectors.

## Material and Methods

### Data collection

DF is a notifiable disease in Australia. Only cases confirmed by clinical and laboratory definitive evidence, based on Australia national notifiable diseases case definitions, are mandated to be reported to The Department of Health^26^. Although the imported dengue cases for visitors are a subset of the imported cases, unfortunately, only data for Australian residents are available in the study. Our study only analyses imported DF cases for Australian residents (Australian citizens and permanent visa holders) returning from overseas. A case is defined as imported when the infected person has a recent travel history from a known dengue endemic region. A patient with no travel history indicates autochthonous transmission. In northern QLD, all cases, whether suspected or confirmed, are referred to the local health authority who implement a system of interviews and vector control that targets local mosquito populations before they can incubate and transmit the virus^27^. This paper focuses only on imported DF cases between 2004–2013 (i.e., it does not include locally acquired DF cases) and those data were provided by the Australian Government’s Department of Health (DoH) from the National Notifiable Diseases Surveillance System^28^. The annual data on ARO and details of the local population (including SEIFA at postcode-level) were based on the 2011 Census in Australia available from the Australian Bureau of Statistics. SEIFA is a continuous variable which characterises, and is positively related to local socio-economic conditions, including wealth, education, occupation and living conditions, and can be used to measure the diversity of social and economic well-being across different regions in Australia^29^.

### Data analysis

#### Spatiotemporal distribution of imported DF cases

Boxplots were used to detect and compare the seasonal variability of monthly imported DF cases across Australia during three periods: 2004–2006, 2007–2009 and 2010–2013. To detect spatiotemporal variation in the imported DF incidence, we mapped the spatial distributions of average annual imported DF incidence at a postal area level in the three study periods across Australia. ArcMap was used to create the maps (Figs 1, 6 and 7) presented in this study^30^. Heat maps of monthly imported DF cases (standardized by the national totals of imported DF cases each year) were generated to compare the spatiotemporal trend of imported DF cases from the different parts of the Pacific region and from Asia, Africa, Europe and the Americas during the three periods.

#### Seasonality

To explore the impact of seasonality on monthly imported DF incidence in Australia, we decomposed monthly imported DF incidence into a seasonal factor series using a seasonal decomposition method. This method decomposes time series data into four new time series in order to reveal any systematic seasonal variation. The model is given by: *Y*_*t*_ = *T*_*t*_ + *S*_*t*_ + *C*_*t*_ + *E*_*t*_, here *Y*_*t*_ denotes the original times series; *T*_*t*_, *S*_*t*_, *C*_*t*_and *E*_*t*_ denote the seasonally adjusted series, the seasonal effect, the trend component and the error component, respectively^31^. *T*_*t*_ is calculated after removing the seasonal variation of the monthly imported DF incidence. *S*_*t*_represents the effect of the seasonal pattern of the monthly imported DF incidence. A simple linear regression model is then built to detect the linear trends in i) the annual number of increased new source countries of DF and ii) the monthly number of locations with imported DF cases.

#### Spatiotemporal model

Moran’s *I* statistic was used to test the spatial autocorrelation among nearby locations across study using ArcMap software^30^. A Bayesian conditional autoregressive (CAR) model^32^ was employed to assess the spatial clusters of mean local imported DF cases after accounting for the effects of local SEIFA across Australia. The advantage of using CAR to analyse the clusters is that it can adjust for confounding factors. Due to the fact that some locations have small populations, we used the Empirical Bayes approach to spatially smooth the observed imported DF incidences to reduce random variation related to small local populations and correct their outlier incidences to obtain adjusted imported DF cases using the GeoDa software^33^,^34^. Thus, letting 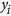 denote the total adjusted imported DF cases in location *i* (*i* = 1…2513), a spatial Poisson model is given by:


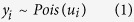



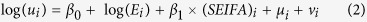


Here ***β*** = (*β*_0_, *β*_1_) is the vector of coefficients for the intercept and SEIFA, respectively; *E*_*i*_ is the expected value of imported DF cases; *μ*_*i*_represents structured heterogeneity and characterizes spatial variation (spatial cluster) in imported DF cases between locations. The CAR model was used to describe *μ*_*i*_ as a function of its neighbours via a normal distribution with conditional weighted means and conditional variance; *v*_*i*_ corresponds to geographically unstructured heterogeneity in imported DF cases. The regression coefficients had diffuse normal priors, given by ***β ***~ *N*(0.0, 1.0E6). The variances of random effects (*μ* and *v*), defined by 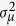 and 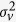, had uniform priors, 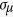 ~ *U*(0, 10) and 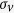 ~ *U*(0, 10). Posterior estimates of parameters were obtained through Markov chain Monte Carlo (MCMC) sampling. Estimation was based on 100,000 iterations after an initial “burn-in” of 20,000 iterations. For Bayesian inference, convergence was assessed by checking the trace and the autocorrelation plots for the sample of the chain. Analyses were undertaken using WinBUGS software version 1.4^35^.

## Additional Information

**How to cite this article**: Huang, X. *et al.* Dynamic spatiotemporal trends of imported dengue fever in Australia. *Sci. Rep.* **6**, 30360; doi: 10.1038/srep30360 (2016).

## References

[b1] Gubler D. J. The global emergence/resurgence of arboviral diseases as public health problems. Archives of medical research 33, 330–342 (2002). 12234522 10.1016/s0188-4409(02)00378-8

[b2] Wilder-Smith A. & Gubler D. J. Geographic expansion of dengue: the impact of international travel. Medical Clinics of North America 92, 1377–1390 (2008). 19061757 10.1016/j.mcna.2008.07.002

[b3] Furuya-Kanamori L. *et al.* Co-distribution and co-infection of chikungunya and dengue viruses. BMC infectious diseases 16, 1 (2016). 26936191 10.1186/s12879-016-1417-2 PMC4776349

[b4] Bhatt S. *et al.* The global distribution and burden of dengue. Nature 496, 504–507 (2013). 23563266 10.1038/nature12060 PMC3651993

[b5] World Health Organization. Dengue guidelines for diagnosis, treatment, prevention and control: new edition, http://www.who.int/tdr/publications/documents/dengue-diagnosis.pdf (Date of access: 6/8/2015) (2009). 23762963

[b6] Messina J. P. *et al.* Global spread of dengue virus types: mapping the 70 year history. Trends in microbiology 22, 138–146 (2014). 24468533 10.1016/j.tim.2013.12.011 PMC3946041

[b7] McBride W. Dengue fever: is it endemic in Australia? Internal medicine journal 40, 247–249 (2010). 20529038 10.1111/j.1445-5994.2010.02196.x

[b8] Russell R. C. *et al.* Dengue and climate change in Australia: predictions for the future should incorporate knowledge from the past. Med J Aust 190, 265–268 (2009). 19296793 10.5694/j.1326-5377.2009.tb02393.x

[b9] Woodruff R. E., McMichael T., Butler C. & Hales S. Action on climate change: the health risks of procrastinating. Australian and New Zealand journal of public health 30, 567–571 (2006). 17209275 10.1111/j.1467-842x.2006.tb00788.x

[b10] Trewin B. J., Kay B. H., Darbro J. M. & Hurst T. P. Increased container-breeding mosquito risk owing to drought-induced changes in water harvesting and storage in Brisbane, Australia. International health 5, 251–258 (2013). 24225151 10.1093/inthealth/iht023

[b11] Kearney M., Porter W. P., Williams C., Ritchie S. & Hoffmann A. A. Integrating biophysical models and evolutionary theory to predict climatic impacts on species’ ranges: the dengue mosquito Aedes aegypti in Australia. Functional Ecology 23, 528–538 (2009).

[b12] Ritchie S., Williams C. R., Russell R. & Sutherst R. Aedes (Stegomyia) albopictus-a dengue threat for southern Australia? Communicable Diseases Intelligence Quarterly Report 29, 296–298 (2005). 16220868 10.33321/cdi.2005.29.31

[b13] Ritchie S. A. *et al.* Discovery of a widespread infestation of Aedes albopictus in the Torres Strait, Australia. Journal of the American Mosquito Control Association 22, 358–365 (2006). 17067032 10.2987/8756-971X(2006)22[358:DOAWIO]2.0.CO;2

[b14] Ritchie S. A. *et al.* Dengue control in north Queensland, Australia: case recognition and selective indoor residual spraying. Dengue Bulletin 26, 7–13 (2002).

[b15] Queensland Government. Queensland dengue management plan 2015–2020, https://www.health.qld.gov.au/publications/clinical-practice/guidelines-procedures/diseases-infection/governance/dengue-mgt-plan.pdf (Date of access: 16/01/2016) (2015).

[b16] Hanna J. N. *et al.* Multiple outbreaks of dengue serotype 2 in north Queensland, 2003/04. Australian and New Zealand Journal of Public Health 30, 220–225 (2006). 16800197 10.1111/j.1467-842x.2006.tb00861.x

[b17] Knope K. & Giele C. & null. Increasing notifications of dengue in Australia related to overseas travel, 1991 to 2012. Communicable diseases intelligence quarterly report 37, E55–E59 (2013). 23692160 10.33321/cdi.2013.37.6

[b18] Chowell G., Cazelles B., Broutin H. & Munayco C. V. The influence of geographic and climate factors on the timing of dengue epidemics in Perú, 1994–2008. BMC infectious diseases 11, 164 (2011). 21651779 10.1186/1471-2334-11-164 PMC3121613

[b19] San Martín J. L. *et al.* The epidemiology of dengue in the Americas over the last three decades: a worrisome reality. The American journal of tropical medicine and hygiene 82, 128–135 (2010). 20065008 10.4269/ajtmh.2010.09-0346 PMC2803522

[b20] Warrilow D., Northill J. A. & Pyke A. T. Sources of Dengue Viruses Imported into Queensland, Australia, 2002–2010. Emerging Infectious Diseases 18, 1850–1857 (2012). 23092682 10.3201/eid1811.120014 PMC3559152

[b21] Jansen C. C. & Beebe N. W. The dengue vector Aedes aegypti: what comes next. Microbes Infect 12, 272–279 (2010). 20096802 10.1016/j.micinf.2009.12.011

[b22] Wilder-Smith A. & Schwartz E. Dengue in travelers. New England Journal of Medicine 353, 924–932 (2005). 16135837 10.1056/NEJMra041927

[b23] Jelinek T., Dobler G., Hölscher M., Löscher T. & Nothdurft H.-D. Prevalence of infection with dengue virus among international travelers. Archives of internal medicine 157, 2367–2370 (1997). 9361578

[b24] Ryan E. T., Wilson M. E. & Kain K. C. Illness after international travel. New England Journal of Medicine 347, 505–516 (2002). 12181406 10.1056/NEJMra020118

[b25] Grange L. *et al.* Epidemiological risk factors associated with high global frequency of inapparent dengue virus infections. Frontiers in immunology 5, e280, doi: 10.3389/fimmu.2014.00280 (2014). PMC4052743 24966859

[b26] Australia Government Department of Health. Australian national notifiable diseases case definitions-Flavivirus infection (unspecified), http://www.health.gov.au/internet/main/publishing.nsf/Content/cda-surveil-nndss-casedefs-cd_flavnec.htm (Date of access: 02/12/2015) (2004).

[b27] Queensland Government. Queensland Dengue Management Plan 2010–2015, https://www.health.qld.gov.au/publications/clinical-practice/guidelines-procedures/diseases-infection/governance/dengue-mgt-plan.pdf (Date of access: 8/12/2015) (2011).

[b28] Australian Government Department of Health. National Arbovirus and Malaria Advisory Committee (NAMAC) annual reports, http://www.health.gov.au/internet/main/publishing.nsf/content/cda-arboanrep.htm (Date of access: 02/09/2015) (2014).

[b29] Australian Bureau of Statistics. Census of Population and Housing: Socio-Economic Indexes for Areas (SEIFA), Australia, 2011 http://www.abs.gov.au/ausstats/abs@.nsf/mf/2033.0.55.001 (Date of access: 5/10/2015) (2013).

[b30] Riley S. Large-scale spatial-transmission models of infectious disease. Science 316, 1298–1301 (2007). 17540894 10.1126/science.1134695

[b31] SPSS Statistics for Windows (2013) [v22] Armonk, NY: IBM Corp. http://www-01.ibm.com/software/au/analytics/spss/.

[b32] Besag J., Green P., Higdon D. & Mengersen K. Bayesian computation and stochastic systems. Statistical Science 10, 3–41 (1995).

[b33] Anselin L. Exploring Spatial Data with GeoDa: A Workbook (Date of access: 21/03/2016). (2005).

[b34] Hu W., Clements A., Williams G. & Tong S. Spatial analysis of notified dengue fever infections. Epidemiology and infection 139, 391–399 (2011). 20392302 10.1017/S0950268810000713

[b35] Spiegelhalter D., Thomas A., Best N. & Lun D. WinBUGS User Manual, http://www.mrc-bsu.cam.ac.uk/bugs/winbugs/manual14.pdf (Date of access: 28/08/2015) (2003).

[b36] ArcGIS (2013) [v10.2] http://www.esri.com/software/arcgis.

